# Management and outcome of prenatal absent pulmonary valve syndrome

**DOI:** 10.1007/s00404-022-06397-4

**Published:** 2022-01-18

**Authors:** Florian Recker, Eva C. Weber, Brigitte Strizek, Annegret Geipel, Christoph Berg, Ulrich Gembruch

**Affiliations:** 1 Department for Obstetrics and Prenatal Medicine, University Hospital Bonn, Venusberg Campus 1, 53127 Bonn, Germany; 2 Division of Prenatal Medicine, Department of Obstetrics and Gynecology, University of Cologne, Cologne, Germany

**Keywords:** APVS, Absent pulmonary valve syndrome, Prenatal diagnosis

## Abstract

**Objective:**

The purpose of this study was to explore the outcome in fetuses with prenatal diagnosis of absent pulmonary valve syndrome (APVS) on ultrasound imaging.

**Methods:**

A manual web scraping technique was utilized, where MEDLINE and EMBASE were searched along the combination with other relevant medical subject headings such as “absent pulmonary valve syndrome”, “prenatal APVS” and “APVS/outcome”. The observed outcomes encompassed the rate of chromosomal abnormalities, associations and malformations linked to APVS and fetuses with APVS. A quality assessment of the included studies was also performed. We used meta-analyses of proportions to combine data and fixed or random-effects models according to the heterogeneity of the results.

**Results:**

Seven studies including 199 fetuses with APVS were included in the analysis. The median gestational age at referral to the tertiary center was 24.8 weeks. An association to tetralogy of Fallot (TOF) could be seen in 84.4% of all cases. In total 140 out of 199 cases underwent invasive testing, with a total number of 55 abnormal karyotypes [39.3% (95% CI 31.1–47.9%)]. 35.2% of the patients opted for termination of pregnancy (95% CI 28.5–42.3%).

**Conclusion:**

The analysis underlines the distribution of fetuses with APVS, with 84.4% of cases presenting with TOF/APVS and only 12.6% having APVS/intact ventricular septum (IVS). Larger and more prospective study analyses is now needed, especially focusing on long-term follow-up periods of fetuses and children with APVS. Particularly as the postnatal course shows great variety depending on prenatal diagnosis.

## Introduction

Absent pulmonary valve syndrome (APVS) is the congenital absence of the pulmonary valve. It is also referred to as pulmonary valve agenesis, because it is the flaw in the tract that causes the restriction of outflow. It is said to be an absent pulmonary valve, because annular stenosis occurs at a varied level along with main pulmonary artery (MPA) dilation. It is possible that symptoms may include dilation of the pulmonary artery and its branches, ventricular septum defect (VSD), and tricuspid atresia. The presence or absence of symptoms varies according to each individual.

APVS occurs in the form of the variant of tetralogy of Fallot with the presence of this problem in 5% of these patients [1].

The APVS is divided into two types by research experts. These consist of (a) APVS with VSD (otherwise called Fallot type APVS) and (b) APVS with intact ventricular septum and the possibility of tricuspid atresia (otherwise called Non-Fallot type APVS). The majority of the cases are of Fallot type APVS [2]. About 0.2–0.4 percent of infants with CHD have Fallot type APVS along with congenital heart disease. The non-Fallot type of APVS rarely occurs and the origin remains unknown.

Tracheobronchial compression occurs as a result of significant aneurysmal dilatation of the pulmonary arteries. There are also increased chances of respiratory failure and bronchomalacia. The patient may suffer from respiratory dysfunction, heart failure, and other serious infections in the postnatal course. After birth, 40–50% of individuals have obstructive ventilation problem indicators, for example: stridor, respiratory failure, tachypnea, and intercostal retractions. These symptoms may sometimes require mechanical ventilation or extracorporeal layer oxygenation (ECMO). In case of a less serious bronchial obstacle, the clinical history points toward the tetralogy of Fallot. Correct diagnosis can reveal the necessity of surgery. Clinical symptoms define the timing of when to conduct surgery, such as patients will be subjected to surgery at the age of 3–6 months if the sign of bronchial compression is absent [3]. Patients who have a large VSD and bronchial compression should be treated sooner before the development of bronchomalacia.

The aim of this systematic search was to analyze prenatal diagnostics in fetuses with APVS to establish how accurate prenatal imaging is in reaching a correct diagnosis and in detecting the presence of associated anomalies.

## Methods

Studies were assessed according to the following criteria: population (*n* > 10), outcome, gestational age at examination and type of imaging assessment of the prenatal absent pulmonary valve syndrome.

Two authors (F.R., U.G.) reviewed all abstracts independently. Agreement regarding potential relevance was reached by consensus and full-text copies of relevant papers were obtained. The same two reviewers independently extracted relevant data regarding study characteristics and pregnancy outcome. Inconsistencies were discussed by the reviewers, or with a third author, and consensus was reached. If more than one study was published for the same cohort with identical endpoints, the report containing the most comprehensive information on the population was included to avoid overlapping populations.

The Newcastle–Ottawa Scale (NOS) for cohort studies was used to assess the quality of the included research. According to the NOS, each study was evaluated based on three key criteria: study group selection, group comparability, and identification of the desired result. The study selection method comprised of identifying the exposed cohort's representativeness, selecting the non-exposed cohort, as well as determining exposure, and showing that the desired outcome did not exist when the study began. But on the other hand, evaluating the comparability of research groups includes determining the comparability of cohorts based on the design or technique. Each numbered item in the selection and result categories received a maximum of one star, according to NOS, whilst comparability received a maximum of two stars.

The search covered both databases of Medline (1950–October 2020) and Embase (1980–October 2020), as well as Cochrane Library and Web of Science with Conference Proceedings (1970–2020). Reference lists of relevant articles were also checked. In addition, the search was not limited by language and auto-alerts in Medline were also run during the course of the review.

All relevant randomized controlled trials (RCTs) or quasi-RCTs were included and due to the small number of RCTs, we also included non-randomized studies (NRSs). Prospective observational studies with controls, retrospective matched-pair studies, and comparative studies from well-defined registries/databases were also included (Table 1).

**Table 1:** Characteristics of included studies of absent pulmonary valve syndrome prenatal diagnosis

Study	Country	Study design	Period analyzed	Cases	Median GA at referral
Axt-Fliedner (2017) [4]	Germany, Belgium, Italy, Ukraine, Poland	Retrospective	2012–2016	71	21.5
Gottschalk (2017) [5]	Germany	Retrospective	2002–2014	40	19.7
Szwast (2014) [6]	USA	Retrospective	2001–2010	21	34
Wertaschnigg (2013) [7]	Canada	Retrospective	2000–2010	12	24
Galindo (2006) [8]	Spain	Retrospective	1998–2004	14	28
Volpe (2004) [9]	Italy	Retrospective	1993–2003	21	24
Razavi (2003) [10]	Great Britain	Retrospective	1988–2000	20	23

### Statistical analysis

Statistical analysis was performed with Stata Version 16.1 (Stata Corp, Lakeway, College Station, Texas, USA). Mean, median, standard deviation (SD), range and 95% confidence intervals (CI) were calculated. Data are shown as *n* (%). Univariate comparisons of dichotomous data were performed using the chi‐square and Fisher's exact tests. Comparisons between groups were performed using the *t* test to test group means with SD. A two‐sided value of *p* < 0.05 was considered statistically significant.

## Results

**Fig. 1:**
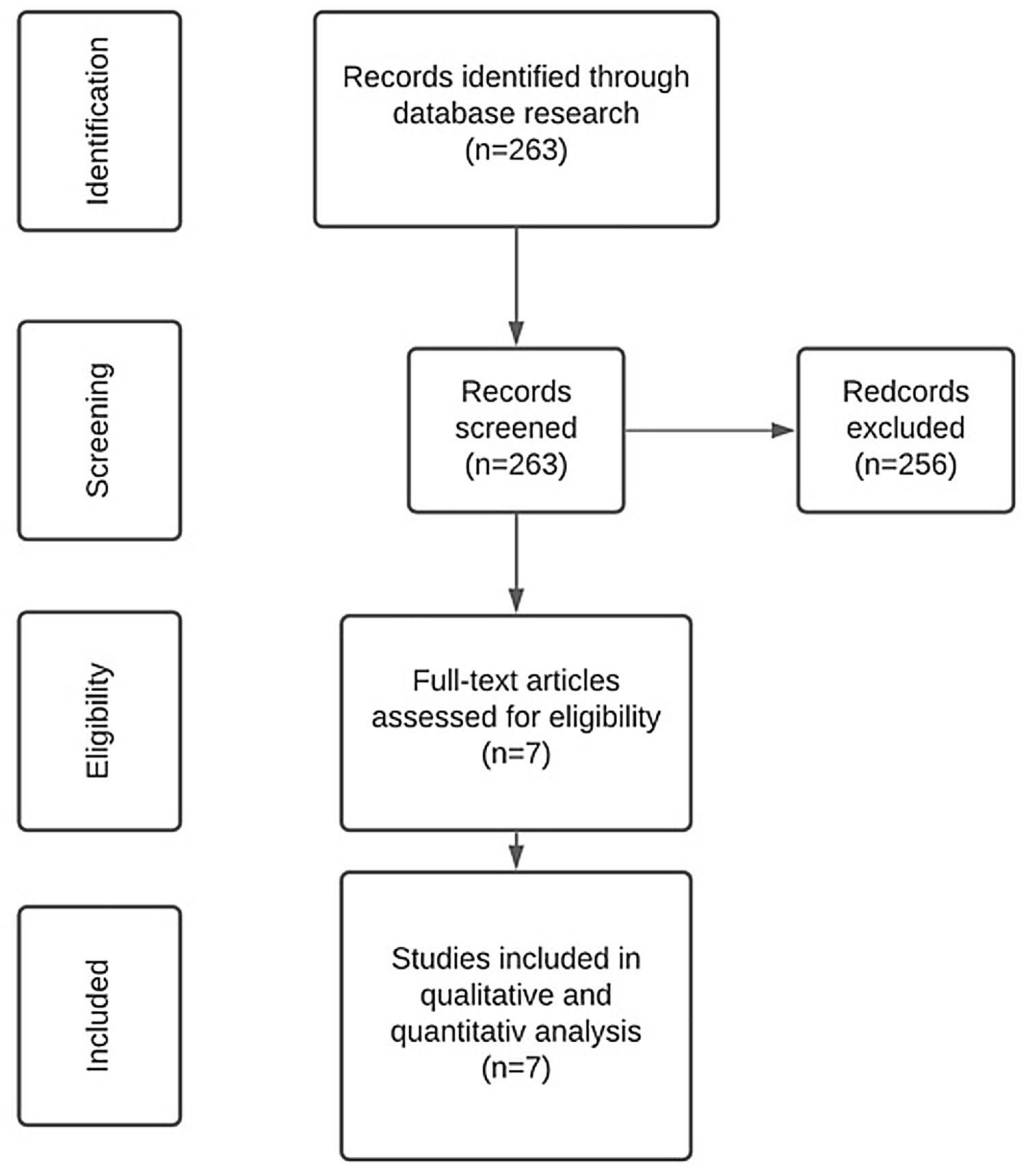
Flowchart summarizing selection of cohort studies on absent pulmonary valve syndrome diagnosed on prenatal ultrasound

A total of 263 articles were identified, of which 7 full-text articles were assessed for their eligibility for inclusion. A total of seven studies were included in the systematic review. The study selection process is outlined in the Preferred Reporting Items for Systematic Reviews and Meta-Analysis (PRISMA) diagram (Fig. 1).

The study was conducted using a retrospective design. The data were collected from the year 1988–2016. A total of 199 fetuses with missing pulmonary valve syndrome were included in the 7 investigations. In six cohort studies, the mean gestational age upon APVS diagnosis was found, whilst the gestational age was absent in one research.

Table 2 shows the findings of the quality assessment of the included studies using the NOS. It is worth mentioning that just the name of the first author is provided. For research group selection and comparability, as well as identifying the target outcome, the majority of the studies included in this evaluation earned excellent scores. However, these studies have severe problems in the retrospective design, sample size, variety of outcomes examined, and varied techniques for prenatal treatment of pregnancies.

**Table 2:** Quality assessment of included studies according to Newcastle–Ottawa Scale for cohort studies

Author	Selection	Comparability	Outcome
Axt-Fliedner (2017)	**	**	**
Galindo (2006)	**	**	**
Gottschalk (2017)	**	**	**
Volpe (2004)	**	**	***
Wertaschnigg (2013)	**	**	***
Razavi (2003)	**	**	***
Szwast (2014)	**	**	***

Only five studies calculated the prevalence of the APVS in the total cohort of congenital heart defects (CDH). The mean prevalence was 1.06% (SD ± 0.34; range 0.80) as a proportion of all prenatally detected CDH. All seven studies reported the median gestational age at referral to the tertiary center, being 24.8 weeks (SD ± 4.77; range 14.30).

**Fig. 2:**
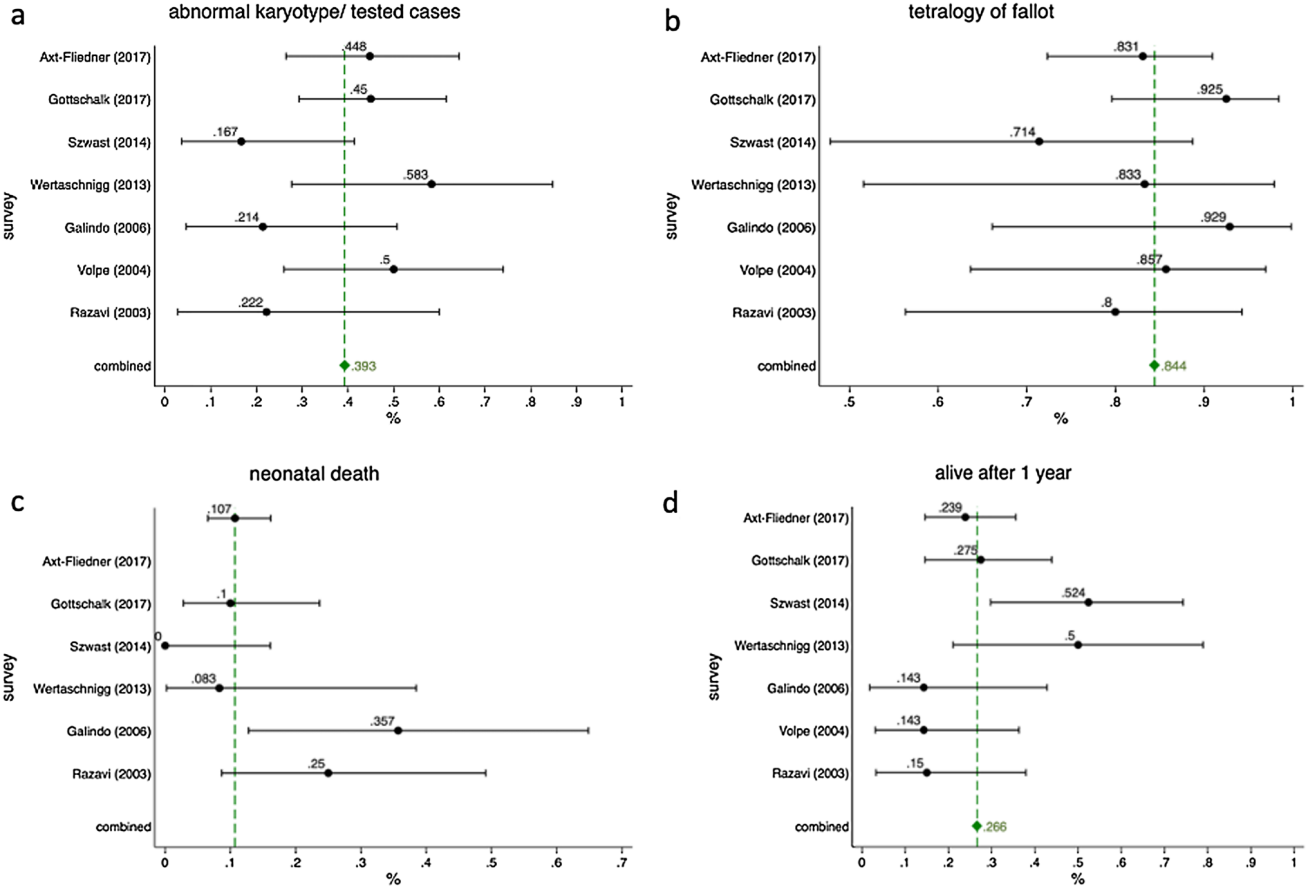
Forest plots of: **a** abnormal karyotype of tested APVS patients; **b** occurrence of tetralogy of Fallot in combination to APVS; **c** neonatal deaths of patients with APVS **d** survival after 1 year with diagnosis of APVS

In 168 fetuses (84.4%) had an associated tetralogy of Fallot and only 25 fetuses (12.6%) had an isolated anomaly with or without a ventricular septum defect. Six patients (3%) had atresia of the tricuspid valve and right ventricular dysplasia. (Fig. 2b). Furthermore, 20 cases (10.1%) showed a right-sided aortic arch.

All seven study cohorts evaluated the occurrence of an abnormal karyotype. In total 140 out of 199 fetuses were tested pre- and postnatally, resulting in 55 abnormal karyotypes [39.3% (95% CI, 31.1–47.9%)] (Fig. 2a). A microdeletion 22q11 was found in the 27 cases with APVS (13.6%). Six studies reported on the outcome of patients with 22q11 microdeletion. Five patients with 22q11 microdeletion survived postnatally (18.5%), whereas 22 patients died during the pregnancy (81.5%). Of these, six patients had a termination of pregnancy (22.2%).

There were 10 cases (5.0%) of trisomy 18 and 11 cases of trisomy 13 (5.5%) reported in the studies. In addition, there was one case found with a deletion of 6q (0.5%), one with Alagille syndrome (0.5%), one with a microdeletion of chromosome 1p3 (0.5%) and one with a microdeletion of 4p1 (0.5%).

Extracardiac abnormalities in all fetuses were not consistently reported in all studies. Reported extracardiac anomalies in fetuses with normal karyotype included hydrops fetalis, mild hydronephrosis, and a labial cyst.

Other extracardiac abnormalities diagnosed in the central nervous system were reported in cases with chromosomal abnormalities (megacisterna magna, hydrocephalus, cleft lip and palate, agenesis of corpus callosum, lissencephaly), in the urogenital system (pyelectasia, bilateral renal agenesis) and in the gastrointestinal system (duodenal atresia). Data reporting concerning nuchal translucency thickness in the seven studies were highly inhomogeneous and only presented in individual studies with an incidence of 40% [8].

All seven studies reported on perinatal outcomes of fetuses with absent pulmonary valve syndrome. In total, 70 parents (35.2%) chose termination of pregnancy (95% CI 28.5–42.3%). Intrauterine fetal demise occurred in 23 cases (11.5%) (95% CI 7.5–16.8%). Furthermore, 6 studies reported on neonatal death which occurred in 19 cases (9%) (95% CI 6.5–16.2). An infant death could be observed in 19 cases (9.5%) (95% CI 6.0–14.5%), whereas 53 children (26.6%) were still alive after 1 year (95% CI 20.6–33.3%) (Fig. 1d).

## Discussion

APVS is characterized by absent pulmonary valve leaflets, stenosis of the pulmonary annulus, and dilatation of the pulmonary artery. The current meta-analysis underlines the distribution of fetuses with APVS, showing 84.4% of cases presenting with TOF/APVS and only 12.6% having APVS/IVS, reflecting previous reports with much lower numbers included.

More than one-third of parents opted for termination of pregnancy. The studies indicate that the extracardiac abnormalities in fetuses with APVS can vary widely. Furthermore, there is no unique clinical picture in prenatal diagnosis when APVS is suspected. Moreover, the diagnosis of APVS was mainly made at 24.8 gestational weeks when referred to the tertiary center.

The strength of this review is in its robust methodology in identifying all possible studies for inclusion, in addition to assessing data quality and synthesizing all suitable data. In this regard, the seven selected studies have substantially comparable criteria permitting the comparison and investigation of prenatal screening for APVS.

Today, the etiology of APVS is still unclear. Becker et al. [11] hypothesized that reduced diastolic pressure due to an absent diastolic closure of the pulmonary valve combined with a nonrestrictive ventricular septal defect, might result in early intrauterine closure of the arterial duct between 14 and 21 gestational weeks. In contrast, some authors have proposed that the underdevelopment of the pulmonary valve leaflets could cause ductal agenesis, or vice versa. Others have postulated that 80–90% of fetuses with APVS and TOF have a DA, as is the case for TOF in general, in addition this subset of fetuses is likely to miscarry early in gestation due to massive aortopulmonary shunting and biventricular overload [12]^13^. Again, the large cohorts available do not conclusively reveal the pathophysiology of APVS. Therefore, the exact pathophysiological mechanism has not been completely elucidated to date.

In this sense, none of the seven publications examined offered significant cohort analysis as the bulk of the research only had a few cases. As a result, it is difficult to make accurate statements regarding the clinical course of APVS. Individual study cohort time durations vary widely and in most situations the cases required longer periods, thus increasing the difficulty to identify the clinical results. Therefore, there can be a significant difference in individual research homogeneity, allowing just a few factors to be compared across individual studies.

## Limitations

Some meta-analyses included only a few trials, and others were restricted to small populations. Moreover, the extraction of individual case data was not possible in some of the articles examined. Routine chromosome analysis may miss small chromosomal abnormalities such as microdeletions. This emphasises the need for molecular cytogenetic methods, which were not employed in the majority of the investigations. As a result, it can be considered that the incidence of chromosomal abnormalities other than aneuploidies were not focused on much in this thorough study.

The investigation of a long-term outcome of the individual cases was missing in several examined studies, whilst data of long-term outcomes were only sparsely and incompletely reported. In a large retrospective work over a 25-year period, Norgaard et al. [14] showed that patients undergoing repair of APVS have a 79% chance of 5-year survival.

Therefore, larger and more prospective study analyses are needed to better examine the follow-up periods of fetuses and children after a prenatal diagnosis of APVS. The postnatal course, in particular, has to be investigated in connection with an early prenatal diagnosis. There is a need for long-term follow-up studies in the future to provide information about the pathophysiology, the prenatal, and clinical outcomes of this disease.

## Data Availability

Not applicable.

## References

[1] Miller RA, Lev M, Paul MH (1962). Congenital absence of the pulmonary valve. The clinical syndrome of tetralogy of Fallot with pulmonary regurgitation. Circulation.

[2] Wu W, Pang K, Lin Q (2015). Echocardiography in the diagnosis of patients with absent pulmonary valve syndrome: a review study of 12 years. Int J Cardiovasc Imaging.

[3] Dorobantu DM, Stoicescu C, Tulloh RM, Stoica SC (2019). Surgical repair of tetralogy of fallot with absent pulmonary valve: favorable long-term results. Semin Thorac Cardiovasc Surg.

[4] Axt-Fliedner R, Kurkevych A, Slodki M (2017). Absent pulmonary valve syndrome—diagnosis, associations, and outcome in 71 prenatally diagnosed cases: APVS—diagnosis, associations and outcome. Prenat Diagn.

[5] Gottschalk I, Jehle C, Herberg U (2017). Prenatal diagnosis of absent pulmonary valve syndrome from first trimester onwards: novel insights into pathophysiology, associated conditions and outcome: absent pulmonary valve syndrome in the fetus. Ultrasound Obstet Gynecol.

[6] Szwast A, Tian Z, McCann M (2014). Anatomic variability and outcome in prenatally diagnosed absent pulmonary valve syndrome. Ann Thorac Surg.

[7] Wertaschnigg D, Jaeggi M, Chitayat D (2013). Prenatal diagnosis and outcome of absent pulmonary valve syndrome: contemporary single-center experience and review of the literature: absent pulmonary valve. Ultrasound Obstet Gynecol.

[8] Galindo A, Gutiérrez-Larraya F, Martínez JM (2006). Prenatal diagnosis and outcome for fetuses with congenital absence of the pulmonary valve. Ultrasound Obstet Gynecol.

[9] Volpe P, Paladini D, Marasini M (2004). Characteristics, associations and outcome of absent pulmonary valve syndrome in the fetus. Ultrasound Obstet Gynecol.

[10] Razavi RS, Sharland GK, Simpson JM (2003). Prenatal diagnosis by echocardiogram and outcome of absent pulmonary valve syndrome. Am J Cardiol.

[11] Becker R, Schmitz L, Guschmann M, Wegner RD, Stiemer B, Entezami M (2001). Prenatal diagnosis of familial absent pulmonary valve syndrome: case report and review of the literature. Ultrasound Obstet Gynecol.

[12] Yeager SB, Van Der Velde ME, Waters BL, Sanders SP (2002). Prenatal role of the ductus arteriosus in absent pulmonary valve syndrome. Echocardiography.

[13] Moon-Grady AJ, Tacy TA, Brook MM, Hanley FL, Silverman NH (2002). Value of clinical and echocardiographic features in predicting outcome in the fetus, infant, and child with tetralogy of Fallot with absent pulmonary valve complex. Am J Cardiol.

[14] Nørgaard MA, Alphonso N, Newcomb AE, Brizard CP, Cochrane AD (2006). Absent pulmonary valve syndrome. Surgical and clinical outcome with long-term follow-up. Eur J Cardiothorac Surg.

